# Near-Infrared Fluorescence Molecular Imaging of Ductal Carcinoma *In Situ* with CD44v6-Specific Antibodies in Mice: A Preclinical Study

**DOI:** 10.1007/s11307-012-0605-8

**Published:** 2012-11-27

**Authors:** Jeroen F. Vermeulen, Aram S. A. van Brussel, Arthur Adams, Willem P. Th. M. Mali, Elsken van der Wall, Paul J. van Diest, Patrick W. B. Derksen

**Affiliations:** 1 Department of Pathology, University Medical Center Utrecht, PO Box 85500, 3508 GA Utrecht, The Netherlands; 2 Department of Radiology, University Medical Center Utrecht, Utrecht, The Netherlands; 3 Division of Internal Medicine and Dermatology, University Medical Center Utrecht, Utrecht, The Netherlands

**Keywords:** Optical imaging, NIRF, DCIS, Breast cancer, Antibody, IRDye800CW, Mouse model

## Abstract

**Purpose:**

The purpose of this study was to develop a molecular imaging technique using tracers specific for ductal carcinoma *in situ* (DCIS) to improve visualization and localization of DCIS during surgery. As CD44v6 is frequently expressed in DCIS, we used near-infrared fluorescently labeled CD44v6-targeting antibodies for detection of DCIS.

**Procedure:**

Mice bearing orthotopically transplanted CD44v6-positive MCF10DCIS DCIS-like tumors and CD44v6-negative MDA-MB-231 control tumors were intravenously injected with IRDye800CW conjugated to CD44v6-specific antibodies or control IgGs. Noninvasive imaging was performed for 8 days postinjection, followed by intraoperative imaging. Antibody accumulation and intratumor distribution were examined.

**Results:**

Maximum accumulation of CD44v6-specific antibodies was obtained 24 h postinjection. Maximum tumor-to-background ratio for MCF10DCIS tumors was 4.5 ± 0.2, compared to 1.4 ± 0.1 (control tumors, *p* = 0.006), and 1.7 ± 0.1 (control IgG, *p* = 0.014), for 8 days postinjection. *Ex vivo*, tumor-to-background ratios were comparable to those obtained by intraoperative imaging.

**Conclusions:**

We show the applicability of noninvasive and intraoperative optical imaging of DCIS-like lesions *in vivo* using CD44v6-specific antibodies.

## Introduction

Molecular imaging of cell surface markers, *e.g.*, growth factor receptors, hypoxia markers, and adhesion molecules, has become an important field for imaging of cancer for diagnosis, assessment of therapy response, or for tumor delineation during surgical resection [1–4]. Achieving radical excision during breast-conserving surgery for ductal carcinoma *in situ* (DCIS), and diffusely growing or small (T1) breast cancers, is challenging, since these lesions are often not palpable. Molecular imaging with near-infrared fluorescent (NIRF) tracers holds promise when applied as a tool for image-guided surgery. First, detection of lesions can be highly sensitive and specific by using targeted tracers. Second, due to its physical properties, NIRF can penetrate several millimeters in tissue, allowing noninvasive visualization of tumors [5]. Third, no ionizing radiation is used, limiting the need for protective measures. Fourth, the spectral properties (emission wavelengths between 700 and 900 nm) of the fluorescent tracers result in low background (auto) fluorescence [6].

Previously, we examined the expression of membrane markers in breast cancer to identify the most sensitive and specific molecular markers for optical imaging. The expression rate of tumor-specific markers did not exceed 20 % of all breast cancers, whereas tumor markers expressed by normal breast epithelium (*i.e.*, with a lower tumor specificity) were expressed in the majority of breast cancers. CD44v6 was expressed in 64 % of breast cancers and thereby the most frequently expressed marker achieving a threefold tumor-to-normal ratio (predefined as sufficient for molecular imaging). Therefore, CD44v6 was considered the most promising tumor marker for molecular imaging of breast cancer [7].

The glycoprotein CD44 is a hyaluronic acid-binding adhesion molecule that facilitates binding of epithelial cells to the extracellular matrix. Due to alternative splicing, CD44 is expressed as multiple isoforms that structurally and functionally differ as a result of changes in the extracellular stem region of the receptor [8, 9]. The standard CD44 variant (CD44s) is widely expressed in epithelial tissues and has been used to mark stem cells, but the expression of these variants is mainly restricted to neoplastic lesions [8–10]. Although the most widely studied variant of CD44, CD44v6, is abundantly expressed in invasive cancers, benign tumors do not express the v6 isoform [10–13]. Despite the high expression in invasive cancer, the relation between aggressiveness, invasiveness, and CD44v6 expression is not clear [14]. A possible role for CD44v6 in tumor progression may lie in its function as co-receptor and scaffolding platform. CD44v6 contains a heparin sulfate side chain able to bind and present glycosylated growth factors to their cognate receptors, thereby potentiating receptor tyrosine kinase signaling [15–19].

In studies investigating radioactively labeled antibodies targeting CD44v6 for detection of head and neck cancer, it was shown that administration was safe and allowed specific tumor detection [20, 21]. Furthermore, imaging of breast cancer with CD44v6 antibodies was only described by one group for detection of T1 cancers with SPECT and showed that 66 % of breast cancers could be correctly assigned [22].

To study the applicability of NIRF-labeled antibodies for noninvasive and intraoperative optical imaging of DCIS *in vivo*, we examined NIRF-labeled CD44v6-specific antibodies in a transplantation-based model of DCIS. Our data indicate that detection of preinvasive lesions with NIRF-labeled antibodies is feasible and not hampered by limited vascularization.

## Methods

### Cell Culture, Virus Generation, and Cell Transduction

MCF10DCIS.com cells (further referred to as MCF10DCIS) were obtained from Asterand Inc. (Detroit, MI, USA) and cultured according to the supplier’s guidelines. MDA-MB-231/Luc^+^ [23] (gift of G. van der Pluijm, Leiden University Medical Center, Leiden, The Netherlands) were cultured in DMEM containing 10 % FCS supplemented with 100 IU/ml penicillin, and 100 μg/ml streptomycin. Both cell lines were confirmed negative for estrogen receptor-α, progesterone receptor, and HER2. CD44v6 and E-cadherin were expressed in MCF10DCIS only. All cell lines were validated by STR analysis and routinely checked for *Mycoplasma* infection. All lines were consistently *Mycoplasma* free.

To generate luciferase-expressing MCF10DCIS cells, pLV-CMV-Luc2-IRES-GFP vector (gift from A. Martens, UMC Utrecht, The Netherlands) was introduced by lentiviral transduction as described before [24]. Transduction efficiency was 100 % (determined by expression of GFP) after two rounds of infection.

### Antibody Production, Fluorescent Labeling, and Binding Affinity Measurements after Labeling

The sequences of the variable domain of the heavy and light chains of humanized VFF18, directed against CD44v6, were obtained from the patent WO2002/094879. DNA of the variable domain fragments was synthesized by GeneArt (Life Technologies, Bleiswijk, The Netherlands). Variable domains were cloned into human IgG expression constructs and produced by U-protein Express (Utrecht, The Netherlands). IgG purification was performed by chromatography on proteinase A columns and eluted with sodium citrate (pH 3.6) followed by desalting and buffer exchange to phosphate-buffered saline (PBS) using the automated AKTA express purifier (GE Healthcare, Hoevelaken, The Netherlands). Protein concentration was determined using a NanoDrop spectrophotometer (Thermo Fisher Scientific, Breda, The Netherlands), and purity was confirmed by Coomassie stain of a SDS-PAGE gel. Human IgG from serum was obtained from Sigma-Aldrich (I4506, Zwijndrecht, The Netherlands) and served as a negative control (further referred to as control IgG). Labeling of IgG antibodies was performed as described before [25]. The NIRF dye IRDye800CW, purchased as an *N*-hydroxysuccinimide ester (LI-COR Biosciences, Lincoln, NE, USA), was incubated in a fourfold molar excess of dye to IgG for 2 h at room temperature. After conjugation, free dye was removed using Zebra Spin Desalting Columns (Thermo Fisher Scientific). Dye-to-protein ratio was determined with the following equation: ${{\mathrm{IR}} \left/ {\mathrm{protein}} \right.}={{{\left( {{{{{A_{774}}}} \left/ {{{\varepsilon_{{\mathrm{IR}\mathrm{Dye}\ 800\mathrm{CW}}}}}} \right.}} \right)}} \left/ {{\left( {{{{{{\mathrm{A}}_{280 }}-\left( {0.03\times {A_{774}}} \right)}} \left/ {{{\varepsilon_{\mathrm{protein}}}}} \right.}} \right)}} \right.}$, where the molar extinction coefficient of IRDye800CW is 240,000 M^−1^ cm^−1^ and the molar extinction coefficient for IgG is 210,000 M^−1^ cm^−1^.

For affinity measurements, 15,000 MDA-MB-231 and MCF10DCIS cells were seeded in 96-well plates (Thermo Fischer Scientific) and allowed to adhere overnight. Next, medium was aspirated, cells were blocked with 4 % Marvel (skimmed milk powder) in PBS, and cells were incubated for 2 h at 4 °C with IRDye800CW-labeled IgG in 2 % Marvel in PBS in the dark. Cells were washed three times with PBS, and bound IgG was detected using an Odyssey Imaging System (LI-COR) at 800 nm. The dissociation constant (*K*_d_) was derived from the concentration of IgG at which half the intensity of *B*_max_ was found. GraphPad Prism 5 software (nonlinear regression—one site-specific binding) was used for computational analyses.

### Mouse Studies

Five-week-old female SCID Beige (C.B-17/IcrHsd-*Prkdc* ^*scid*^ *Lyst* ^*bg*^) immunodeficient mice (Harlan Laboratories, Horst, The Netherlands) were orthotopically transplanted as described before [24], with some modifications. Approximately 4 × 10^4^ luciferase-expressing MCF10DCIS and 1 × 10^5^ luciferase-expressing MDA-MB-231 cells were injected using a 10-μl Hamilton syringe in the fourth (inguinal) and third (thoracic) mammary fat pad, respectively. Tumor growth was monitored on a weekly basis using bioluminescence imaging (PhotonIMAGER, Biospace Lab, Paris, France). Upon development of palpable tumors (typically 2–3 mm in diameter), mice were intravenously injected in the tail vein with 100 μg fluorescently labeled IgG. All animal experiments were approved by the Utrecht University Animal Experimental Committee (DEC-Utrecht no. 2011.III.03.027).

### Imaging and Image Analysis

Probe distribution was visualized and quantified based on the fluorescent signal from the labeled CD44v6 and control IgGs. A real-time intraoperative multispectral fluorescence imaging system, developed by the group of Ntziachristos *et al.*, was used for the measurements [26]. In summary, the system consists of a charge-coupled digital iXon3 DU888 camera (Andor Technology, Belfast, UK), cooled at −80 °C for sensitive fluorescence signal detection, and a continuous wave laser with an excitation wavelength of 750 nm for optimal excitation of IRDye800CW. The following imaging parameters were used: distance between object and lens 30–32 cm, zoom 43 %, focus 0 %, and iris 93 %. The exposure time for each image was set at 150 ms and gain at 1,000. The field of view for each image was 125 × 125 mm, corresponding to a resolution of 0.25 × 0.25 mm per pixel. Static images were acquired every 30 min in the first 2 h postinjection and subsequently 3, 4, and 8 h postinjection. After the first day, images were acquired daily until 8 days postinjection.

After image acquisition, a region of interest (ROI) was drawn around each tumor, and the average signal intensity was determined. For each time point, the same size of the ROI was used. Also, an equal-sized ROI was drawn in a representative region without tumor tissue to determine background fluorescence levels and to be able to calculate tumor-to-background ratios. All values are displayed as mean ± standard error of the mean (SEM).

### Biodistribution of IRDye800Cw-Labeled Antibodies

One week postinjection of the CD44v6 or control IgGs, mice were sacrificed and organs were collected, weighted, and snap-frozen in liquid nitrogen and stored at −80 °C. Tissues and tumors were homogenized in RIPA buffer supplemented with protease inhibitors using a TissueLyser II system (QIAGEN, Venlo, The Netherlands). A dilution series of homogenized organs was made in order to measure the intensity in the linear range at 800 nm with the Odyssey Imager (LI-COR). The quantity of IRDye800CW was determined by intra- and extrapolation of the fluorescent value from a calibration curve that consisted of serial dilutions of the injected probe as described before [25, 27].

### Immunohistochemistry

Immediately after resection, the tumors were fixed in neutral buffered formalin, paraffin-embedded, and stored in the dark. Immunohistochemistry was performed on 4-μm-thick sequential sections. Following deparaffinization and rehydration, endogenous peroxidase activity was blocked for 15 min in buffer solution containing 0.3 % hydrogen peroxide. The different antigen retrieval methods used were as follows: boiling for 20 min in 10 mM citrate pH 6.0 (CD44v6), Tris/EDTA pH 9.0 (p63), or pepsin (1 mg/ml) for 15 min at 37 °C (human IgG). A cooling period of 30 min preceded the primary antibody incubation: p63 (clone 4A4, Neomarkers) 1:400, human IgG specific for gamma chains (A0423, DAKO, Glostub, Denmark) 1:500, or CD44v6 (clone VFF18, BMS125 Bender MedSystems, Vienna, Austria) 1:500. The signal was amplified using BrightVision poly-HRP anti-mouse/rabbit/rat (DPVO-HRP, Immunologic, Duiven, The Netherlands) and developed with diaminobenzidine, followed by counterstaining with hematoxylin, dehydration in alcohol, and mounting. Appropriate negative and positive controls were used throughout. For detection of IRDye800CW, tumor slides were deparaffinized, mounted with Immu-mount (Thermo Fisher Scientific), and scanned using the Odyssey Imaging System.

### Statistics

Statistical analysis was performed using IBM SPSS Statistics version 18.0 (SPSS Inc., Chicago, IL, USA). Comparison of tumor to background levels of injected probes was performed using Mann–Whitney *U* test. Wilcoxon signed-rank test was performed to compare the fluorescent intensity of noninvasive with intraoperative imaging. *p* values <0.05 were considered to be statistically significant.

## Results

### Characterization of CD44v6 Antibodies for Noninvasive Imaging of Breast Cancer

The potential of CD44v6-specific antibodies (further referred to as CD44v6 Ab) as tracer for optical imaging was examined in a model for preinvasive breast cancer. Labeling efficiency, expressed as IRDye800CW-to-protein ratio, was 1.43 and 1.57 for CD44v6 Ab and human serum IgG (further referred to as control IgG), respectively. After purification, 5.6 % free dye remained present, which was comparable to previous studies [25]. The apparent affinity (*K*_d_) of labeled CD44v6 Ab was 10 nM and 17 μM on MCF10DCIS (CD44v6 positive) and MDA-MB-231 (CD44v6 negative) cells, respectively. Control IgG had an apparent affinity of approximately 40 nM (MCF10DCIS) and 90 nM (MDA-MB-231), but with maximum binding (*B*_max_) 13 times smaller than CD44v6 Ab on MCF10DCIS cells.

**Fig. 1:**
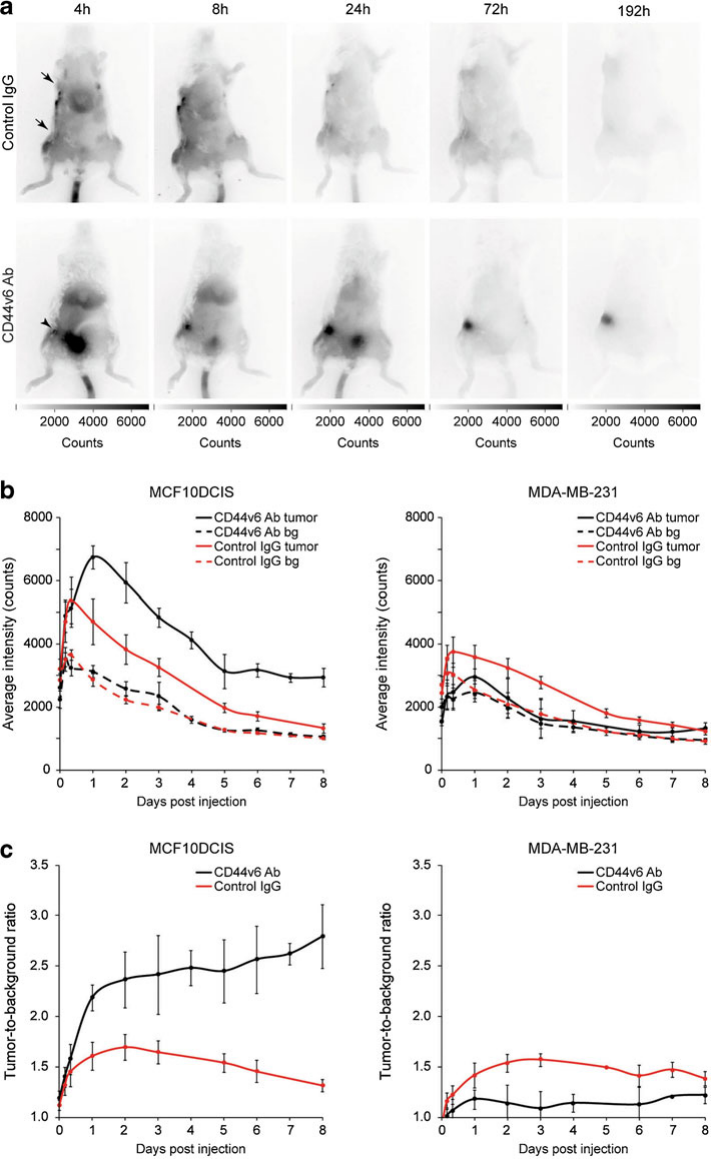
Noninvasive optical molecular imaging of breast cancers. **a** Representative SCID Beige mice bearing orthotopically transplanted MCF10DCIS (inguinal) and MDA-MB-231 (thoracic) tumors. Mice were intravenously injected in the tail vein with CD44v6 Ab or control IgG. At 4 h postinjection, tumor accumulation of CD44v6 Ab was observed in the MCF10DCIS tumors (*arrowhead*), whereas no accumulation of control IgG was observed in MCF10DCIS or MDA-MB-231 tumors (arrows). **b** Fluorescence intensity of MCF10DCIS tumors (*left panel*) or MDA-MB-231 tumors (*right panel*) and background of mice injected with CD44v6 Ab or control IgG over time. Data are displayed as average ± SEM (*n* = 6). (*bg* = background). **c** Tumor-to-background ratio of CD44v6 Ab and control IgG in MCF10DCIS and MDA-MB-231 tumors. Data are displayed as average ± SEM (*n* = 6).

Mice bearing MCF10DCIS and MDA-MB-231 tumors (used as a CD44v6-negative control) were intravenously injected with IRDye800CW-conjugated CD44v6 Ab or control IgG. Accumulation of CD44v6 Ab in the MCF10DCIS tumor became detectable 4 h postinjection, whereas control IgG was not (Fig. 1a). A clear signal of the MCF10DCIS tumor was obtained from 3 days onwards, due to accumulation of the tracer in the tumor and decreased background signal from circulating tracer. In contrast, accumulation of free IRDye800CW was not observed (data not shown), while levels of control IgG were similar in MCF10DCIS *vs.* MDA-MB-231 tumors (Fig. 1a). The maximal fluorescence intensity in the MCF10DCIS tumor was reached after 8 h (control IgG) and 24 h (CD44v6 Ab) and decreased to background levels in 8 days (control IgG) or stabilized after 5 days (CD44v6 Ab) (Fig. 1b). These differences are most likely caused by dissimilar pharmacokinetics of the antibodies used. Fluorescence intensity of control IgG and CD44v6 Ab in the MDA-MB-231 control tumor was lower than the MCF10DCIS tumor, while the background levels and the decrease in fluorescent signal were comparable (Fig. 1b). As a result, tumor-to-background ratio for CD44v6 Ab increased from 2.41 ± 0.39 3 days postinjection to 2.78 ± 0.31 7 days postinjection and tended to increase further in MCF10DCIS (Fig. 1c). In contrast, tumor-to-background ratio of control IgG declined to 1.31 ± 0.06 8 days postinjection and was significantly lower than CD44v6 Ab (*p* = 0.004) in the MCF10DCIS tumor. The tumor-to-background ratio of CD44v6 Ab in the MDA-MB-231 tumor was comparable to control IgG (1.41 ± 0.11, *p* = 0.201) and significantly lower than in the MCF10DCIS tumor (*p* = 0.011).

Because the antibody levels 8 days postinjection were relatively low and positioning of the mice and localization of the tumor could influence the accuracy of the fluorescent signal levels obtained (and thus the tumor-to-background ratio), we determined the coefficient of variation of the optical imaging technique. Four mice were imaged four times each, with readjustment of the imaging device, repositioning of the mice, and reassessing the volume and location of the ROI. The coefficient of variation was 6.1 %, supporting the reproducibility of the optical imaging technique.

### Performance of the Intraoperative Camera System for Noninvasive Imaging

**Fig. 2:**
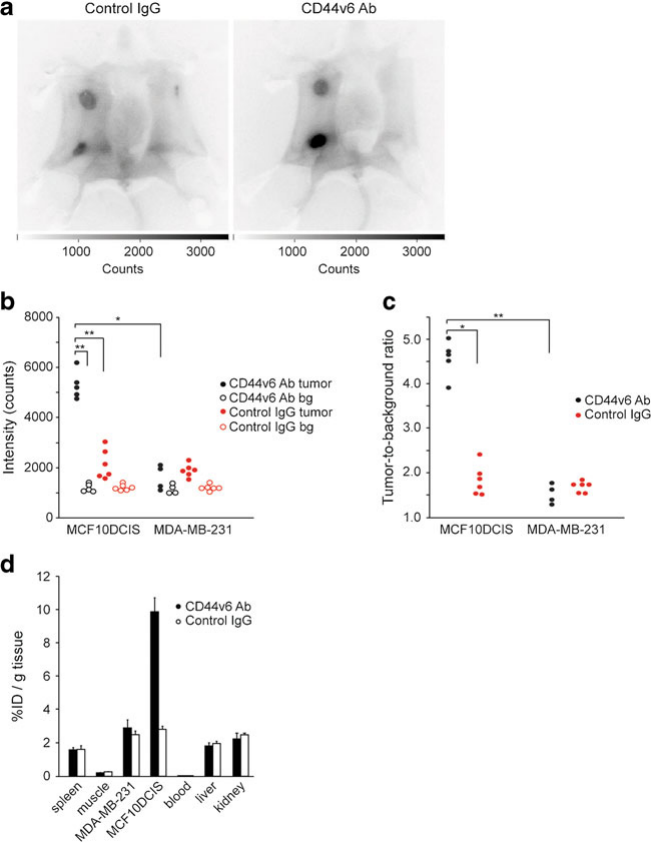
Intraoperative imaging of breast cancers. **a** Representative intraoperative fluorescence images of mice bearing MDA-MB-231 tumors and MCF10DCIS tumors 8 days postinjection with control IgG and CD44v6 Ab. Clear accumulation of CD44v6 Ab was observed in the MCF10DCIS tumor compared to control IgG. Higher signals in both tumors compared to the background were found, independent of the injected antibody due to enhanced perfusion and retention of the tumor. **b** Fluorescence intensity of MCF10DCIS and MDA-MB-231 tumors and the corresponding background (*bg*) in individual mice (*p < 0.05; **p < 0.01). **c** Tumor-to-background ratio of CD44v6 Ab and control IgG in MCF10DCIS and MDA-MB-231 tumors displayed for individual mice (*p < 0.05; **p < 0.01). **d** Biodistribution of CD44v6 Ab and control IgG 8 days postinjection. Tissue levels are expressed as percentage injected dose per gram tissue (*%ID/g*) as average ± SEM (*n* = 6).

Since the camera system used is intended for intraoperative image-guided surgery rather than for noninvasive imaging and quantification of tracer accumulation, we examined whether the performance differed between these applications. Noninvasive imaging was performed on day 8, directly followed by intraoperative imaging, using identical imaging parameters. Specific accumulation of the CD44v6 Ab was observed in the MCF10DCIS tumor (Fig. 2a). As expected, we neither detected accumulation of the CD44v6 Ab in the MDA-MB-231 tumor nor was control IgG observed in the MCF10DCIS tumor. Tumor intensity of MCF10DCIS and MDA-MB-231 tumors compared to the surrounding tissue (skin and abdomen) was higher, independent of the injected IgG (Fig 2a, b), most likely due to probe retention caused by enhanced tumor vascularization. MCF10DCIS tumor signals were significantly higher in CD44v6 Ab-injected mice intraoperatively, compared to the MDA-MB-231 tumors in the same mouse and compared to the MCF10DCIS tumors in mice injected with control IgG (*p* = 0.014 and *p* = 0.006, respectively; Fig. 2b). Accordingly, the resulting tumor-to-background ratios were significantly higher for CD44v6 Ab in MCF10DCIS *vs.* MDA-MB-231 (4.5 ± 0.18 *vs.* 1.4 ± 0.11, *p* = 0.006) and for CD44v6 Ab *vs.* control IgG in the MCF10DCIS tumor (4.5 ± 0.18 *vs.* 1.7 ± 0.05, *p* = 0.014), indicating specific accumulation of CD44v6 Ab in the MCF10DCIS tumors (Fig. 2c).

To test the performance of the imaging system, we quantified all MCF10DCIS tumors using noninvasive and intraoperative imaging after CD44v6 Ab treatment. Compared to intraoperative imaging, fluorescence intensity of MCF10DCIS lesions with noninvasive imaging was significantly lower in CD44v6 Ab-injected (5,083 *vs.* 2,900 counts, *p* = 0.043) and in IgG-injected mice (2,135 *vs.* 1,537 counts, *p* = 0.028). In conclusion, NIRF intraoperative imaging yields a superior tumor-to-background ratio compared to noninvasive imaging.

### Biodistribution of NIRF-Labeled Antibodies

To quantitate the tumor uptake of CD44v6 and control IgG, liver, kidneys, blood, spleen, and muscle of individual mice were collected directly after intraoperative imaging. Eight days postinjection, the levels of CD44v6 and control IgG in blood were very low (2 ‰ of the injected dose (ID) per gram tissue) (Fig. 2d). The levels of IgG in muscle was low (approximately 0.2 % ID/g tissue), while 9.9 ± 0.8 % ID/g of the CD44v6 Ab and 2.8 ± 0.2 % ID/g tissue of the control IgG were present in the MCF10DCIS tumor. The percentage of the injected dose per gram tissue of the CD44v6 Ab or the control IgG in the MDA-MB-231 tumor (2.9 ± 0.5 *vs.* 2.5 ± 0.3 % ID/g, respectively) was slightly higher compared to spleen, kidney, and liver. Furthermore, no difference was found between the injected dose per gram tissue of control IgG in the MDA-MB-231 and MCF10DCIS tumors (Fig. 2d). In conclusion, our data indicate that NIRF signals measured with intraoperative imaging (8 days postinjection) reflect the actual levels of NIRF tracer in the tumors and can be used as a surrogate measure for biodistribution.

### Heterogeneous CD44v6 Antibody Uptake in Preinvasive Breast Cancer Lesions

**Fig. 3:**
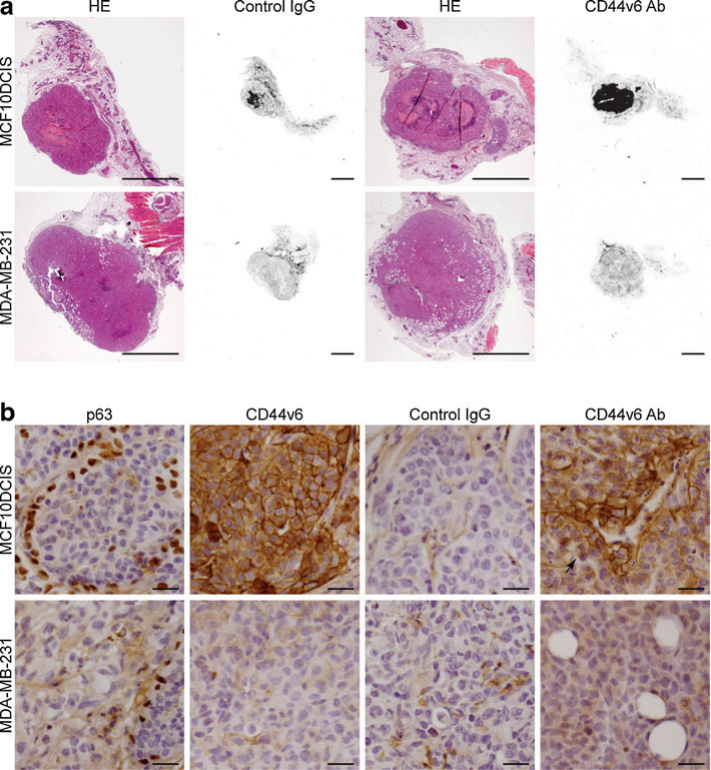
Tumor-specific accumulation of fluorescent tracers in breast cancer. **a** Representative sections of MDA-MB-231 tumors and MCF10DCIS tumor of mice injected with control IgG or CD44v6 Ab. Hematoxylin and eosin (*HE*)-stained sections show the noninvasive character of the MCF10DCIS tumor maintaining the preexisting ductal structures intact, whereas the MDA-MB-231 tumors are invading in the mammary fat pad. Accumulation of IRDye800CW-labeled antibodies was detected for CD44v6 Ab in the MCF10DCIS tumor (fluorescence in control IgG was caused by necrosis for unknown reasons). *Size bar* equals 2 mm. **b** Immunohistochemical characterization of breast cancers and evaluation of intratumor distribution of injected antibodies. The noninvasive (DCIS) phenotype of MCF10DCIS lesions was shown by p63 staining of the myoepithelial cells surrounding the MCF10DCIS lesion, which was absent in the MDA-MB-231 tumor. CD44v6 was homogeneously expressed in the MCF10DCIS lesion, which correlated with tumor accumulation of injected CD44v6 Ab. In addition, no accumulation of control IgG was observed in the MCF10DCIS and MDA-MB-231 lesions, whereas CD44v6 Ab accumulation in MCF10DCIS lesions was mainly restricted to the first two cell layers aligning blood vessels or stroma (*arrow*). *Size bars* equal 50 μm.

As shown in Fig. 3a, no accumulation of control IgG was observed in both MDA-MB-231 and MCF10DCIS tumors, while CD44v6 Ab specifically accumulated in the MCF10DCIS lesion, which is in line with the imaging results. Similar to human DCIS, p63 staining of the myoepithelial cells surrounding the MCF10DCIS lesion confirmed the noninvasive phenotype of the MCF10DCIS lesion. Staining for the injected tracers by immunohistochemistry revealed a clear difference in tumor distribution; low levels of control IgG were present in the stroma surrounding the tumor cells (Fig. 3b), while CD44v6 Ab was exclusively bound to the epithelial cells of the MCF10DCIS lesion and correlated with CD44v6 expression. Furthermore, staining suggested that tumor penetration of CD44v6 Ab was limited to the first two cell layers aligning blood vessels or stroma (Fig. 3b). These results show that tumor penetration of antibodies is limited and resulted in heterogeneous tumor distribution. Further, detection of DCIS-like lesions using NIRF tracers was not hampered by the noninvasive phenotype of the DCIS, suggesting that molecular imaging is suitable for detection of DCIS *in vivo*.

## Discussion

There is increasing interest in molecular imaging of breast cancer. Multiple membrane markers, *e.g.* growth factor receptors and hypoxia-upregulated membrane markers, are currently investigated as candidates for molecular imaging of breast cancer [4]. We showed previously that CD44v6 might be required as tumor marker to achieve sufficient sensitivity for molecular imaging, since growth factor receptors and hypoxia-upregulated membrane markers alone are too infrequently expressed in breast cancer [7].

In the present study, we show that optical imaging with IRDye800CW-labeled humanized antibodies directed to CD44v6 is feasible in a model of preinvasive breast cancer. We could assess the specific uptake of the tracer *in vivo* and demonstrate application of this tracer for intraoperative surgery purposes. Tumor accumulation of IRDye800CW-labeled CD44v6 antibodies in our study (9.9 ± 0.8 % ID/g) was comparable to studies using radiolabeled CD44v6 antibodies, which reported a tumor accumulation of 12.9–15.4 % ID/g in human or 15.3 % ID/g using A431 xenografts in mice [21, 28]. This indicates that biodistribution of IRDye800CW-labeled CD44v6-specific antibodies is comparable to radiolabeled CD44v6 antibodies, which was also recently shown for epidermal growth factor receptor (EGFR)-specific antibodies [27]. In comparison with previous studies using bevacizumab and trastuzumab as tracers for optical imaging of breast cancer (tumor-to-background ratios of 1.93 ± 0.40 and 2.92 ± 0.29, respectively) [29], the tumor-to-background ratios of CD44v6 Ab were higher after 6–8 days. Whether these differences were caused by differences in pharmacokinetics or related to target expression remains unclear. Furthermore, using a control IgG, we were able to determine the contribution of perfusion/nonspecific accumulation to the tumor-to-background ratio, which attributed as much as 50 % to the tumor-to-background ratio in the first 2 days.

In the present study, noninvasive imaging underestimated fluorescence signal intensities of the tumors by approximately 50 %, which might be caused by absorption of fluorescence signal by the skin and the subcutaneous and mammary fat. This directly affected the minimal tumor size we could detect, *i.e.*, DCIS lesions of approximately 3 mm were detectable *in vivo*, while submillimeter DCIS lesions could be detected with intraoperative imaging. Therefore, noninvasive imaging of breast cancer may significantly underestimate the tumor size, tumor uptake, and/or tumor-to-background ratios of injected tracers. More importantly, it might even falsify the conclusions drawn regarding the suitability of a tracer for molecular imaging. Given these potential disadvantages of optical imaging, detection of small breast cancer and *in situ* lesions in patients (*e.g.*, when molecular imaging is applied for screening purposes) might become problematic due to limited excitation power of the laser, localization of the breast tumor, absorption by breast tissue/tumor, and size of the breast. Upcoming clinical trials with IRDye800CW-conjugated antibodies will demonstrate the value of molecular optical imaging for screening purposes.

We showed previously that within normal breast epithelium, myoepithelial cells express low levels of CD44v6. Therefore, the choice of CD44v6 as an imaging target might result in increased background signal from normal breast epithelium and thereby diminished sensitivity and specificity for detection of breast cancer or DCIS lesions. In our preclinical model, the normal (mouse) mammary epithelium did not express CD44v6 and thus did not influence the specificity and sensitivity of detection. Although the uptake of the normal human breast tissue was comparable to that of tumors, Koppe *et al.* showed that while SPECT imaging of T1 tumors using CD44v6 antibodies had sufficient sensitivity to detect the majority of breast cancers due to increased cellularity of the tumor tissue, the limited resolution of the camera was likely hampering the detection of the cancer in the remaining patients. In addition, when less than 20 % of tumor cells were positive for CD44v6, SPECT imaging was not able to detect the lesion [22]. For optical imaging methods, the influence of heterogeneous target expression on the tumor detection is not described, but our unpublished data reveal a similar pattern using optical imaging.

Another parameter attenuating imaging sensitivity was intratumor distribution of the tracers. We found that diffusion of IgGs from tumor-associated blood vessels was limited to the aligning first two to three cell layers of the lesion, probably due to size-limited diffusion. Tumor accumulation, expressed as injected dose per gram tissue of CD44v6 Ab after 8 days, was not different from previous studies performed with Erbitux after 24 h [25]. This suggests that maximum tumor accumulation is achieved 1 day postinjection and that increased tumor-to-background ratios are solely achieved by clearance of circulating antibodies. Increasing tumor accumulation by size reduction of the tracers could enhance the sensitivity of optical imaging for small breast cancers and DCIS. In the case of EGFR, improved tumor uptake and intratumor distribution were achieved by using VHHs (15-kDa antibody fragments consisting of only the Vh domain of the heavy-chain-only antibodies from camelids). In the study of Oliveira *et al.*, VHH-based tracers showed a maximum uptake after 2 h resulting in homogeneous distribution, suggesting a better tumor penetration [25]. For detection of DCIS and other poorly vascularized lesions, application of VHHs and other small tumor-specific tracers for optical imaging might be the preferred option for optical imaging.

## Conclusions

Using CD44v6-specific antibodies, we show that near-infrared optical molecular imaging has sufficient sensitivity for noninvasive and intraoperative imaging of DCIS lesions *in vivo*. This opens the way to clinical image-guided surgery trials in humans. In parallel, further improvements may be achieved by better tumor penetration through size reduction of tracers.
